# Fixation stability and implant-associated complications in periacetabular osteotomy: a comparison of screw and K-wire fixation

**DOI:** 10.1007/s00402-021-04112-7

**Published:** 2021-08-18

**Authors:** Vincent Justus Leopold, Juana Conrad, Robert Karl Zahn, Christian Hipfl, Carsten Perka, Sebastian Hardt

**Affiliations:** Department of Orthopaedic Surgery and Traumatology, Charité Berlin, University Hospital Berlin, Chariteplatz 1, 10117 Berlin, Germany

**Keywords:** Developmental dysplasia of the hip, Periacetabular osteotomy, Surgical technique, Fixation options

## Abstract

**Aims:**

The aim of this study was to compare the fixation stability and complications in patients undergoing periacetabular osteotomy (PAO) with either K-wire or screw fixation.

**Patients and methods:**

We performed a retrospective study to analyze a consecutive series of patients who underwent PAO with either screw or K-wire fixation. Patients who were treated for acetabular retroversion or had previous surgery on the ipsilateral hip joint were excluded. 172 patients (191 hips: 99 K-wire/92 screw fixation) were included. The mean age at the time of PAO was 29.3 years (16–48) in the K-wire group and 27.3 (15–45) in the screw group and 83.9% were female. Clinical parameters including duration of surgery, minor complications (soft tissue irritation and implant migration) and major complications (implant failure and non-union) were evaluated. Radiological parameters including LCE, TA and FHEI were measured preoperatively, postoperatively and at 3-months follow-up.

**Results:**

Duration of surgery was significantly reduced in the K-wire group with 88.2 min (53–202) compared to the screw group with 119.7 min (50–261) (*p* < 0.001). Soft tissue irritation occurred significantly more often in the K-wire group (72/99) than in the screw group (36/92) (*p* < 0.001). No group showed significantly more implant migration than the other. No major complications were observed in either group. Postoperative LCE, TA and FHEI were improved significantly in both groups for all parameters (*p* = < 0.0001). There was no significant difference for initial or final correction for the respective parameters between the two groups. Furthermore, no significant difference in loss of correction was observed between the two groups for the respective parameters.

**Conclusion:**

K-wire fixation is a viable and safe option for fragment fixation in PAO with similar stability and complication rates as screw fixation. An advantage of the method is the significantly reduced operative time. A disadvantage is the significantly higher rate of implant-associated soft tissue irritation, necessitating implant removal.

**Level of evidence:**

III, retrospective trial.

## Introduction

Periacetabular osteotomy (PAO) is an established technique in the treatment of developmental dysplasia of the hip (DDH) in young adults [1–3].With the aim of preventing or at least delaying development of secondary osteoarthritis, excellent results both clinically and radiologically have been described in short-term, mid-term and long-term follow-up [1–4].

The surgical technique has been well described [1]. During PAO, the acetabulum is completely released from the pelvis through five osteotomies resulting in free movement and the possibility of three-dimensional reorientation of the hip socket [5]. In the original description of the procedure, fixation of the reoriented acetabular fragment is achieved through screw fixation [1, 5]. According to the current literature, screw fixation is the standard procedure in fragment fixation in PAO. But despite good clinical results, non-union remains a common complication [6–8]. The literature shows that with increasing rigidity of the fragment fixation, the rates of non-union also increase [9–11]. In a study by Clohisy et al., screw fixation was performed and 8% non-unions were observed [10]. In another study by Clohisy et al., screw and plate fixation was combined and the rate of non-union increased to 19% [9]. As an alternative, semi-rigid Kirschner wire (K-wire) fixation of the acetabular fragment combined with allogenous bone grafting has been described showing good results with a reduced rate of non-union [11]. The idea behind this technique is to combine a semi-rigid fixation with a stable press-fit allograft allowing minimal movement of the acetabulum, but enough stability to prevent a loss of correction. With less fixation rigidity, one could assume that a loss of correction is more likely. However, comprehensive studies comparing K-wire fixation and screw fixation regarding stability and complications are lacking.

This study aims to assess the fixation stability and rate of implant-associated complications of K-wire fixation compared to screw fixation after acetabular reorientation in PAO.

## Materials and methods

### Demographics

After obtaining approval from the local ethics committee, we performed a retrospective study of 172 consecutive patients (191 hips) undergoing PAO between January 2015 and June 2017 with a primary diagnosis of acetabular dysplasia. Inclusion criteria were patients with adequate radiological imaging pre- and postoperatively and at least 3 months follow-up. Exclusion criteria were patients who were treated with PAO for indications other than symptomatic DDH such as acetabular retroversion and patients who had prior surgery on the ipsilateral hip joint, leaving a final cohort of 172 patients (191 hips). Mean follow-up was at 94 days (SD 12.3; range 70–112).

All hips showed at least one radiologic abnormality, including lateral center edge angle of Wiberg (LCE) less than 25° [12], acetabular inclination (AI) greater than 10° [12], an anterior center–edge angle (ACE) as described by Lequesne and de Seze of less than 25° [13] and a femoral head extrusion index (FHEI) as described by Heyman and Herndeon of greater than 26% [14]. Femoral head congruency was determined by 30° abduction functional radiographs preoperatively and was good in all hips. Demographic data collected included age, gender, body mass index (BMI) and duration of surgery measured from skin incision until the completion of skin closure. Demographics are shown in Table 1.

**Table 1:** Demographics: values are presented as mean, standard deviation (SD) and range (*unpaired *t* test; **Fisher’s exact test)

	K-wire fixation (99 hips)	Screw fixation (92 hips)	*p* values
Age	29.24 (SD 7.07; range 16–48)	27.34 (SD 8.60; range 15–45)	0.098*
BMI	23.88 (SD 4.27; range 16.9–35.0)	24.63 (SD 4.59; range 16.3–35.9)	0.307*
Male/female	11/87	20/72	0.075**
Follow-up (days)	94.18 (SD 11.05; range 76–107)	86.40 (SD 12,11; range 72–106)	0.073*

### Surgical technique

**Fig. 1:**
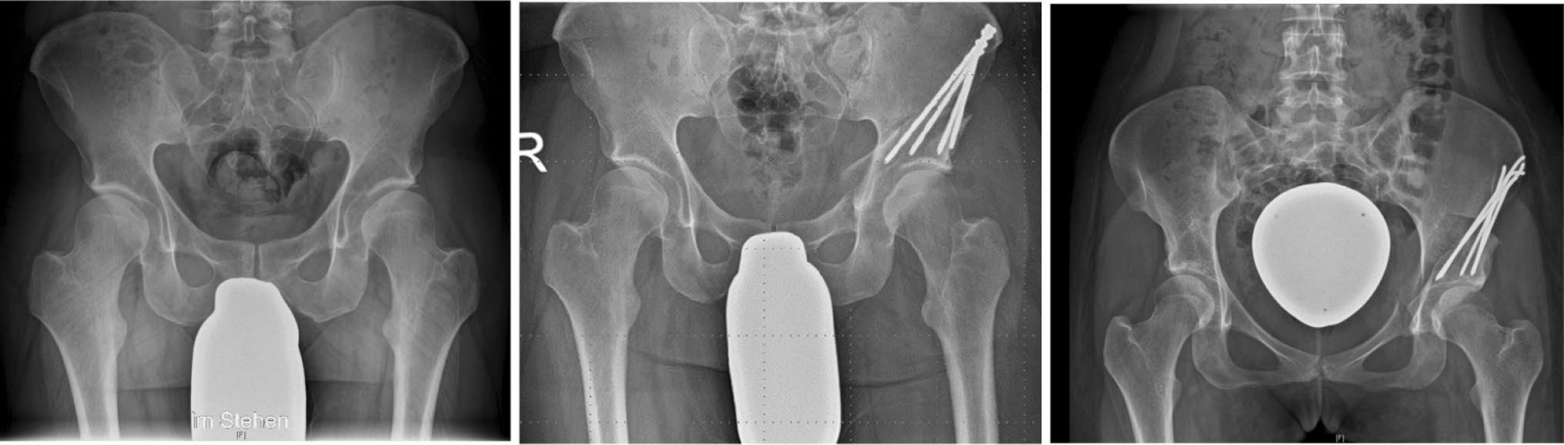
**a** Preoperative radiographic assessment for indication of PAO. **b** Correction and fixation with screws. **c** Correction and fixation with K-wires

All PAOs were performed by two experienced orthopedic surgeons at our institution with one of them using K-wire fixation (*n* = 99) and the other screw fixation (*n* = 92) (Fig. 1).

The surgical technique of PAO has been described previously [1]. In the presented study, acetabular reorientation and fixation was achieved under fluoroscopic guidance using an anterior approach as described in the original description of the PAO. Fixation of the acetabular fragment was achieved through fixation of either three to four screws (4.5 mm) or four to five unthreaded K-wires (2.5 mm) introduced through the iliac crest. Additionally, allogenic bone grafts were induced into the supra- and retroacetabular gap previously created by iliac osteotomy as previously described [11]. The target of intraoperative reorientation was defined as normalization of LCE greater than 25°, AI less than 10° and FHEI between 10 and 26%.

All patients of both groups were mobilized in the same way using a standard mobilization regimen allowing tip-touch partial weight-bearing of the operated extremity for the first 6 weeks postoperatively. Weight-bearing was then increased to half of the patient’s body weight from the 7th to the 10th postoperative week and gradual increase to full weight-bearing thereafter until 3 months postoperatively. No limitation to range of motion of the hip joint was imposed.

### Radiological assessment and complications

**Fig. 2:**
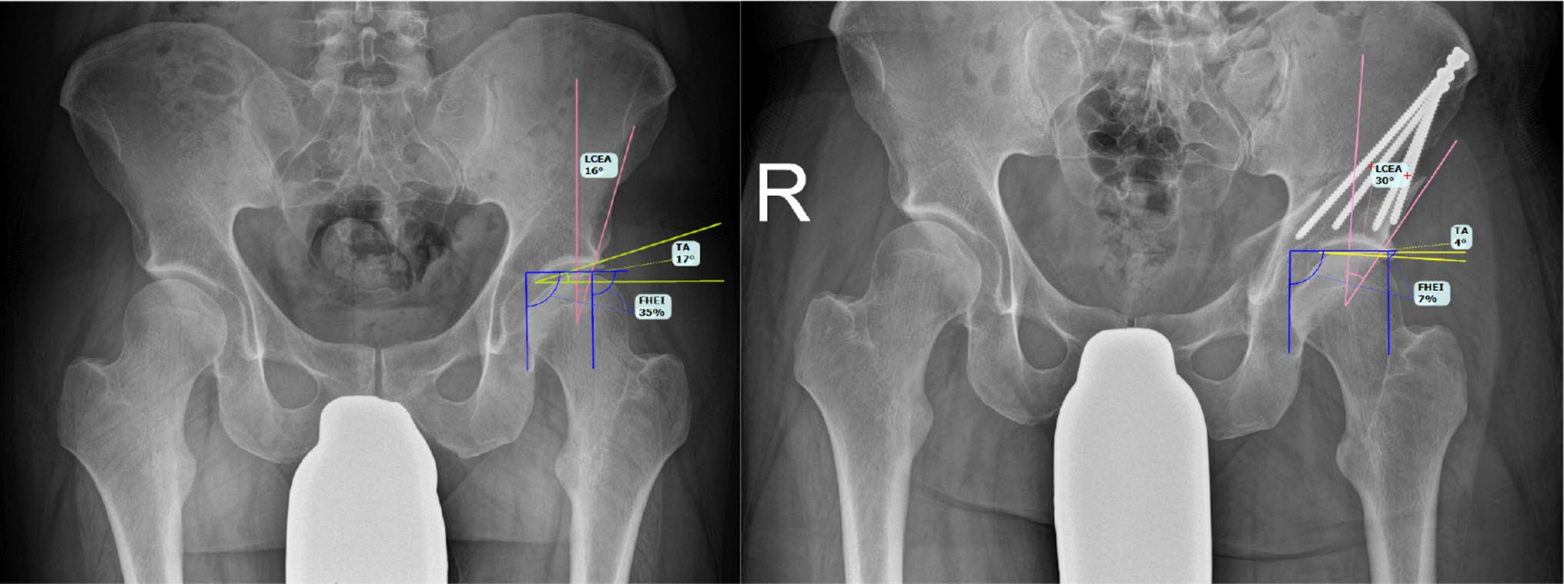
Radiological measurement of lateral center edge angle (LCEA), Tönnis angle (TA) and femoral head extrusion index (FHEI) on **a** preoperative and **b** postoperative a.p. pelvis X-rays

All patients received standardized standing AP pelvis radiographs. Radiological parameters relevant for DDH were measured preoperatively, postoperatively before discharge and at 3 months follow-up: lateral center edge angle (LCEA), Tönnis angle (TA) as well as femoral head extrusion index (FHEI). An example of the measurement of radiological parameters is shown in Fig. 2. Osseous bone healing was determined through absence or presence of consolidation across the osteotomies in a.p. and axial radiographs. All measurements were performed by two residents (VL, JC), both trained by the same senior orthopedic surgeon (SH). A loss of correction was defined as the difference between initial correction at immediate postoperative time and at 3 months follow-up. A clinically significant loss of correction was defined as a loss of the acetabular fixation requiring revision surgery or a delta LCEA of more than 5° as measured in the radiological assessment.

Intra- and postoperative complications were reviewed. Minor complications were defined as soft tissue irritation requiring implant extraction surgery or implant migration of more than 5 mm. Major complications were defined as implant failure, intraarticular implant migration or non-union.

### Statistical analysis

Intra- and interrater reliability was assessed using an intraclass correlation coefficient (ICC) model. Kappa value was used to confirm intra- and interrater reliability. Frequency rates, means and range were utilized to describe baseline patient characteristics. Normal distribution was tested using the Shapiro–Wilk-test. *T* test was used to determine significant differences between continuous data and Chi-square for categorical data. A *p* value of less than 0.05 was considered statistically significant. For documentation of the collected data, Microsoft Excel version 16.16.2 was used. The collected data were analyzed using IBM SPSS 25.

## Results

### Radiological assessment

The inter- and intra-observer reliabilities for the measurements of radiological parameters were excellent for all parameters, ranging from 0.974 to 0.989. For preoperative and postoperative measurements, see Table 2. The postoperative LCEA, TA and FHEI were improved significantly in both the K-wire and the screw group for all parameters (*p* = < 0.0001). The initial corrections for LCEA (*p* = 0.789), TA (*p* = 0.312) and FHEI (*p* = 0.786) were not significantly different between the K-wire group and the screw group. The final correction of the respective parameters was also not significantly different between both groups for LCEA (*p* = 0.551), TA (*p* = 0.307) and FHEI (*p* = 0.854). The loss of correction showed no significant difference between the K-wire and screw group for LCEA (*p* = 0.234), TA (*p* = 0.272) and FHEI (*p* = 0.854). An overview of the measured radiological parameters is shown in Table 2. Overall, no case with a clinically significant loss of correction as measured by the radiographs or leading to reoperation was observed in either group.

**Table 2:** Comparison of correction of LCE, TA and FHEI between the two groups; values are presented as mean, standard deviation (SD) and range (*unpaired *t* test)

	K-wire group (99 hips)	Screw group (92 hips)	*p* values
LCEA (°)			
Preoperative	15.24 (SD 6.44; range − 3 to 26)	17.58 (SD 6.08; range − 11 to 26)	0.011*
Postoperative	28.89 (SD 6.33; range 8 to 43	30.84 (SD 6.18; range 7 to 42)	0.040*
Initial correction	13.70 (SD 6.65; range 0 to 34)	13.45 (SD 6.29; range − 2 to 39)	0.789*
Final correction	14.50 (SD 6.69; range 1 to 30)	13.62 (SD 6.97; range − 4 to 43)	0.551*
Loss of correction	− 0.20 (SD 2.15; range − 4 to 4)	− 0.48 (SD 2.92; range − 6 to 9)	0.234*
Tönnis angle (°)			
Preoperative	14.81 (SD 7.21; range 2 to 37)	11.62 (SD 6.32; range − 2 to 32)	0.001*
Postoperative	3.68 (SD 7.73; range − 19 to 26)	− 0.67 (SD 6.19; range − 14 to 13)	0.001*
Initial correction	− 11.12 (SD 7.03; range − 32 to 14)	− 12.16 (SD 7.13; range − 31 to 7)	0.312*
Final correction	− 10.94 (SD 7.43; range − 25 to 12)	− 12.51 (SD 7.02; range − 31 to 7)	0.307*
Loss of correction	− 0.08 (SD 2.46; range − 5 to 6)	− 0.66 (SD 2.44; range − 7 to 7)	0.272*
FHEI (%)			
Preoperative	25.02 (SD 10.16; range 4.87 to 54.14)	21.59 (SD 8.39; range 0.13 to 59.24)	0.012*
Postoperative	10.02 (SD 9.48; range − 7.27 to 53.91)	8.27 (SD 7.52; range − 8.38 to 27.90)	0.162*
Initial correction	− 14.99 (SD 7.84; range − 48.07 to 0.15)	− 13.31 (SD 7.15; range − 40.32 to 1.81)	0.125*
Final correction	− 13.60 (SD 10.96; range − 39.02.− 4.10)	− 13.04 (SD 8.64; range − 40.32 to 17.69)	0.786 *
Loss of correction	− 0.95 (SD 4.88; range − 10 to 15)	0.73 (SD 6.06; range − 18.90 to 21.55)	0.854*

Bold values indicate significance

### Operating time and complications

The operating time was significantly lower in the K-wire group 88.2 min (53–202) compared to the screw group 119.7 (50–261) (*p* < 0.001).

A significant difference in the incidence of implant-associated soft tissue irritation was observed between the investigated groups. In the K-wire group, 72 out of 99 hips had soft tissue irritation and were therefore admitted to implant extraction surgery compared to 36 out of 92 hips in the screw group (*p* < 0.0001). Implant migration as defined above was observed in 0 out of 99 hips in the screw-fixation group compared to 4 out of 92 hips in the K-wire fixation group showing no significant difference between both groups (*p* = 0.121). Both groups showed no case of non-union, implant failure or intraarticular implant migration.

## Discussion

DDH is a complex pathology of the hip and the leading cause of secondary osteoarthritis of the hip [15]. If diagnosed in time and correctly indicated, PAO is a good option in operative therapy of DDH in the adult showing good to excellent outcomes [2, 4, 16–18].

Through three-dimensional reorientation and fixation of the acetabulum, better coverage of the femoral head is provided. So far, most previous studies described the use of screw fixation. It is assumed that this rigid fixation is necessary to provide and maintain stability and thus secure consolidation of the osteotomies [1, 4, 18, 19]. The idea behind fragment fixation with K-wires is that combining a semi-rigid fixation with a stable press-fit allograft allows minimal movement of the acetabulum, yet still provides enough stability to prevent a loss of correction.

To our knowledge, this is the first study to compare the stability of K-wire- and screw fixation in PAO.

The most important finding of the presented study is that there is no clinically significant loss of correction with the investigated modified technique using K-wire fixation compared to screw fixation. It can therefore be assumed that even if semi-rigid fixation is used, sufficient stability is achieved to obtain the correction result. Even a more restrictive postoperative treatment in the sense of prolonged partial weight-bearing did not seem necessary according to the results of our cohort, since all patients were mobilized according to the same postoperative mobilization regimen.

A previous study investigated the occurrence of non-unions in PAO with K-wire fixation. However, detailed radiological evaluation of loss of correction was not reported in that study [6]. Other studies comparing different screw-fixation techniques and their stability have been published numerously. These studies involved the biomechanical comparison of different fixation models in PAO. [20–23]. For example, Babis et al. were able to show higher stability in more rigid fixation constructs in an in vitro model [23]. Widmer et al. were also able to show higher stability for more rigid fixations [21]. However, these studies were in vitro models with simulated forces similar to or even exceeding those of full weight-bearing. Furthermore, none of these studies investigated the stability of K-wire fixation in PAO.

K-wire fixation as an alternative to screw fixation has previously been reported in pelvic surgery. It is successfully used for fixation of pelvic osteotomies in children and adolescents and also, for example, in pelvic osteotomies for Perthes disease [24]. However, the conditions in these cases can only be applied to the biomechanical conditions in PAO in adults to a limited extent. Biomechanical studies comparing fixation with K-wires and screws in complex multidimensional pelvic osteotomies are lacking.

Our study involved in vivo conditions using a mobilization regimen with postoperative partial weight-bearing. Postoperative mobilization with partial weight-bearing is regularly used after PAO and is also described in the literature by the first describers of PAO [1, 5].

In terms of complications, the K-wire group showed significantly more implant-associated soft tissue irritations requiring implant removal compared to the screw-fixation group. In our opinion, this is caused by the relatively bigger portion of osteosynthesis material protruding from the bone of the iliac crest in an area with relatively little soft tissue coverage. A previous study found similar complication potential in their study without further quantifying or comparing it [11]. Thawrani et al. described a similar incidence of soft tissue irritation in their study of complications of the established technique with screw fixation [25], similar to the screw group investigated in this study. On the one hand, retained implants may pose a problem for MRIs that may be necessary in the future, as metal implants cause artifacts and thus reduce the quality of the imaging [26].

On the other hand, retained implants may pose a challenge for conversion to THA, which is still necessary in a relevant proportion of patients [4].

There was no significant difference in implant failure between the two groups. No major complications such as non-union or intraarticular implant migration were found in either group. Even though in our study little minor and no major complications can be reported, PAO is still a surgical method in which higher complication rates were frequently described. Overall complication rates in previous studies vary between 1 and 86% for major complications and 11 and 100% for minor complications [11, 25].

The K-wire group had significantly reduced surgery time compared to the screw group. The fact that the two surgeons each used one of the fixation techniques results in a certain bias and the reduced operation time cannot be attributed with certainty to the choice of fixation. However, from our point of view it seems logical that the operation time can be reduced if the intermediate step of changing the temporary fixation with K-wires to the definitive fixation with screws is omitted if no screws are used, whereas in screw fixation this intermediate step is regularly performed [1]. This could be an advantage insofar as various studies have shown that prolonged duration of surgery is a risk factor for perioperative complications in orthopedic surgery as well as in other surgical fields [27, 28]. Tahwrani et al. found that prolonged surgery time in PAO is a risk factor for major complications as well as blood loss in PAO and therefore needs to be avoided as much as possible [25].

This study has several limitations. First, it has a retrospective study design. Second, preoperative measurements of the relevant radiological parameters show more dysplastic values in the K-wire group. However, both groups were corrected to almost the same extent and the desired correction was achieved as shown in the postoperative measurements in both groups. Third, in our cohort the procedure was performed by two different surgeons. Fourth, 3 months follow-up is relatively short. However, we believe that with painless full weight-bearing and radiologically proven bony consolidation already achieved at this time, further loss of correction thereafter seems unlikely.

This study demonstrated that K-wire fixation is a viable and safe option in fragment fixation in PAO with similar stability and complication rates compared to screw fixation. An advantage of the method is the significantly reduced operating time. A disadvantage is the significantly higher rate of implant-associated soft tissue irritation requiring implant extraction. Further studies are needed to examine the presented surgical technique at a long-term follow-up. Furthermore, biomechanical studies could provide further information about the stability of the investigated method. These findings can be taken into account by surgeons when choosing the form of fixation of the acetabular fragment in PAO.

### Take home message

**Table:** 

K-Wire fixation is a viable and safe option for fragment fixation in PAO with comparable results regarding fixation stability and complication rates. An advantage of the investigated method is the significantly reduced operative time. A disadvantage is the significantly higher rate of implant-associated soft-tissue irritation necessitating implant removal.	

## References

[1] Ganz R, Klaue K, Vinh TS, Mast JW (1988). A new periacetabular osteotomy for the treatment of hip dysplasias. Technique and preliminary results. Clin Orthop Relat Res.

[2] Wells J, Schoenecker P, Duncan S, Goss CW, Thomason K, Clohisy JC (2018). Intermediate-term hip survivorship and patient-reported outcomes of periacetabular osteotomy: the Washington University experience. J Bone Jt Surg Am.

[3] Maranho DA, Williams KA, Millis MB, Kim YJ, Novais EN (2018). Mid-Term results of periacetabular osteotomy for the treatment of hip dysplasia associated with Down syndrome: minimum follow-up of five years. J Bone Jt Surg Am.

[4] Lerch TD, Steppacher SD, Liechti EF, Tannast M, Siebenrock KA (2017). One-third of hips after periacetabular osteotomy survive 30 years with good clinical results, no progression of arthritis, or conversion to THA. Clin Orthop Relat Res.

[5] MaRG W (2002). Die Berner periazetabuläre Osteotomie. Oper Orthop Traumatol.

[6] Matta JM, Stover MD, Siebenrock K (1999). Periacetabular osteotomy through the Smith-Petersen approach. Clin Orthop Relat Res.

[7] Biedermann R, Donnan L, Gabriel A, Wachter R, Krismer M, Behensky H (2008). Complications and patient satisfaction after periacetabular pelvic osteotomy. Int Orthop.

[8] Peters CL, Erickson JA, Hines JL (2006). Early results of the Bernese periacetabular osteotomy: the learning curve at an academic medical center. J Bone Jt Surg Am.

[9] Clohisy JC, Nunley RM, Curry MC, Schoenecker PL (2007). Periacetabular osteotomy for the treatment of acetabular dysplasia associated with major aspherical femoral head deformities. J Bone Jt Surg Am.

[10] Clohisy JC, Barrett SE, Gordon JE, Delgado ED, Schoenecker PL (2006). Periacetabular osteotomy in the treatment of severe acetabular dysplasia. Surgical technique. J Bone Jt Surg Am.

[11] Wassilew GI, Janz V, Renner L, Perka C, Pruss A (2016). Reduced rates of non-union with modified periacetabular osteotomy using peracetic-acid sterilized cancellous allografts. Cell Tissue Bank.

[12] Massie WK, Howorth MB (1950). Congenital dislocation of the hip. Part I. Method of grading results. J Bone Jt Surg Am.

[13] Lequesne M, De S (1961). False profile of the pelvis. A new radiographic incidence for the study of the hip. Its use in dysplasias and different coxopathies. Rev Rhum Mal Osteoartic.

[14] Heyman CH, Herndon CH (1950). Legg–Perthes disease; a method for the measurement of the roentgenographic result. J Bone Jt Surg Am.

[15] Ipach I, Mittag F, Syha R, Kunze B, Wolf P, Kluba T (2012). Indications for total hip arthroplasty in young adults—idiopathic osteoarthritis seems to be overestimated. Rofo.

[16] Mayo KA, Trumble SJ, Mast JW (1999). Results of periacetabular osteotomy in patients with previous surgery for hip dysplasia. Clin Orthop Relat Res.

[17] Trousdale RT, Ekkernkamp A, Ganz R, Wallrichs SL (1995). Periacetabular and intertrochanteric osteotomy for the treatment of osteoarthrosis in dysplastic hips. J Bone Jt Surg Am.

[18] Clohisy JC, Schutz AL, St John L, Schoenecker PL, Wright RW (2009). Periacetabular osteotomy: a systematic literature review. Clin Orthop Relat Res.

[19] Siebenrock KA, Schoeniger R, Ganz R (2003). Anterior femoro-acetabular impingement due to acetabular retroversion. Treatment with periacetabular osteotomy. J Bone Jt Surg Am.

[20] Yassir W, Mahar A, Aminian A, Newton P, Wenger D (2005). A comparison of the fixation stability of multiple screw constructs for two types of pelvic osteotomies. J Pediatr Orthop.

[21] Widmer BJ, Peters CL, Bachus KN, Stevens PM (2010). Initial stability of the acetabular fragment after periacetabular osteotomy: a biomechanical study. J Pediatr Orthop.

[22] Kashima N, Shiramizu K, Nakamura Y, Moriyama S, Naito M (2015). Biomechanical comparison of the fixation after curved periacetabular osteotomy using titanium and bioabsorbable screws. Hip Int.

[23] Babis GC, Trousdale RT, Jenkyn TR, Kaufman K (2002). Comparison of two methods of screw fixation in periacetabular osteotomy. Clin Orthop Relat Res.

[24] Westhoff B, Krauspe C, Lederer R (2019). Morbus Perthes – Neuigkeiten in der Diagnostik und Behandlung. Perthes disease—news in diagnostics and treatment. Der Orthopäde.

[25] Thawrani D, Sucato DJ, Podeszwa DA, DeLaRocha A (2010). Complications associated with the Bernese periacetabular osteotomy for hip dysplasia in adolescents. J Bone Jt Surg Am.

[26] Zou YF, Chu B, Wang CB, Hu ZY (2015). Evaluation of MR issues for the latest standard brands of orthopedic metal implants: plates and screws. Eur J Radiol.

[27] Jaffer AK, Barsoum WK, Krebs V, Hurbanek JG, Morra N, Brotman DJ (2005). Duration of anesthesia and venous thromboembolism after hip and knee arthroplasty. Mayo Clin Proc.

[28] Clarke-Pearson DL, DeLong ER, Synan IS, Coleman RE, Creasman WT (1987). Variables associated with postoperative deep venous thrombosis: a prospective study of 411 gynecology patients and creation of a prognostic model. Obstet Gynecol.

